# Rapid Detection of Trichodysplasia Spinulosa-Associated Polyomavirus in Skin Biopsy Specimen

**DOI:** 10.1128/genomeA.00694-14

**Published:** 2014-07-24

**Authors:** Paulo Roberto P. Urbano, Cláudio Sérgio Pannuti, Ligia C. Pierrotti, Elias David-Neto, Camila Malta Romano

**Affiliations:** a Instituto de Medicina Tropical de São Paulo e Departamento de Moléstias Infecciosas e Parasitárias (LIM-52), Universidade de São Paulo, São Paulo, Brazil; b Departamento de Moléstias Infecciosas, Faculdade de Medicina, Universidade de São Paulo, São Paulo, Brazil; c Departamento de Urologia, Serviço de Transplante Renal do Hospital das Clínicas da Faculdade de Medicina, Universidade de São Paulo, São Paulo, Brazil

## Abstract

Trichodysplasia spinulosa-associated polyomavirus (TSV) is responsible for a rare skin cancer. Using metagenomic approaches, we determined the complete genome sequence of a TSV first detected in Brazil in spicules of an immunocompromised patient suspected to have trichodysplasia spinulosa.

## GENOME ANNOUNCEMENT

Trichodysplasia spinulosa-associated polyomavirus (TSV) was identified in 2010 in a patient suffering from trichodysplasia spinulosa (TS), a rare skin disease characterized by the development of follicular papules and keratin spicules (1). Trichodysplasia spinulosa (TS) is very rare and has a similar pattern among affected patients, demonstrating hair follicle dilatation and keratotic plugging of the infundibulum and keratin spicules, which is most prominent in the central facial region (2–4). As with other polyomaviruses, TSV has a circular genome, and the orientation and size of its open reading frames (ORFs) (VP1, VP2, VP3, and T-antigen [LT-ag]), as well as the presence of a noncoding control region (NCCR) in between, are similar to those of the other members from this family (5).

In 2013, a skin biopsy specimen suspected to be infected with TS was received in the Laboratory of Virology of the Institute of Tropical Medicine in São Paulo for diagnostic workup. The patient was an immunocompromised renal recipient presenting spicules in the central facial region resembling those observed during classical TS presentation. In order to investigate the presence of TSV and any putative DNA virus, we performed a viral metagenomic analysis on this sample. The initial treatment with DNase was performed in the macerate tissue to remove all DNA-free molecules, leaving only the encapsulated intact virus. The sample was then directly sequenced in the Ion Torrent platform.

*De novo* assembly of the reads revealed the presence of trichodysplasia spinulosa virus in the sample. The full genome is 5,235 nucleotides long, and a similarity analysis revealed that the virus found in Brazil is the most divergent among the four TSV genomes already reported from Japan, the Netherlands, and the United States. While the genetic distance (*D*) between Brazilian TSV and United States/Netherland/Japan TSV was around 0.008, *D* values of 0.004 and 0.0009 were obtained from comparisons between viruses from Japan and the United States-Netherlands, and the United States and the Netherlands, respectively. Using sequences from the Netherlands and the United States as references, the Brazilian TSV presented a total of 45 polymorphisms (42 single nucleotide substitutions [SNPs] and 3 indels). The NCCR region comprises 11 SNPs and all identified indels. One specific nucleotide change (G to C at position 5118) is located in a GC-rich region near a binding site for LT-ag, GCCTCT**G**/**C** (where underlining indicates the LT-ag binding site and boldface indicates the nucleotide change) (6). The VP1 gene presented six nucleotide changes, VP2 had three (two of them also in the VP3 frame), and LT-ag showed 16 SNPs. The only nonsynonymous change was found in LT-ag, at site 349 (I to T) of the amino acid sequence.

Over the past few years, several human polyomaviruses have been discovered through new sequencing technologies. Nevertheless, few studies were dedicated to the functional and molecular characterization of these new members, and much remains unknown. Here, we report the fifth TSV complete genome, which is also the first detection of this virus made in South America.

### Nucleotide sequence accession number.

The genomic sequence of the TSV Brazilian strain has been deposited in GenBank under the accession no. KM007161.
